# The influence of cancer tissue sampling on the identification of cancer characteristics

**DOI:** 10.1038/srep15474

**Published:** 2015-10-22

**Authors:** Hui Xu, Xin Guo, Qiang Sun, Mengmeng Zhang, Lishuang Qi, Yang Li, Libin Chen, Yunyan Gu, Zheng Guo, Wenyuan Zhao

**Affiliations:** 1 College of Bioinformatics Science and Technology, Harbin Medical University, Harbin, 150086, China; 2 Genomics Research Center, Harbin Medical University, Harbin, 150086, China; 3 Key Laboratory of Ministry of Education for Gastrointestinal Cancer, Department of Bioinformatics, Fujian Medical University, Fuzhou, 350004, China

## Abstract

Cancer tissue sampling affects the identification of cancer characteristics. We aimed to clarify the source of differentially expressed genes (DEGs) in macro-dissected cancer tissue and develop a robust prognostic signature against the effects of tissue sampling. For estrogen receptor (ER)+ breast cancer patients, we identified DEGs in macro-dissected cancer tissues, malignant epithelial cells and stromal cells, defined as Macro-Dissected-DEGs, Epithelial-DEGs and Stromal-DEGs, respectively. Comparing Epithelial-DEGs to Stromal-DEGs (false discovery rate (FDR) < 10%), 86% of the overlapping genes exhibited consistent dysregulation (defined as Consistent-DEGs), and the other 14% of genes were dysregulated inconsistently (defined as Inconsistent-DEGs). The consistency score of dysregulation directions between Macro-Dissected-DEGs and Consistent-DEGs was 91% (P-value < 2.2 × 10^−16^, binomial test), whereas the score was only 52% between Macro-Dissected-DEGs and Inconsistent-DEGs (P-value = 0.9, binomial test). Among the gene ontology (GO) terms significantly enriched in Macro-Dissected-DEGs (FDR < 10%), 18 immune-related terms were enriched in Inconsistent-DEGs. DEGs associated with proliferation could reflect common changes of malignant epithelial and stromal cells; DEGs associated with immune functions are sensitive to the percentage of malignant epithelial cells in macro-dissected tissues. A prognostic signature which was insensitive to the cellular composition of macro-dissected tissues was developed and validated for ER+ breast patients.

Macro-dissected cancer tissues contain both carcinoma cells and stromal cells with distinct gene expression patterns^1^, and tissue sampling for gene expression profiling experiments commonly requires that the proportion of carcinoma cells is greater than certain threshold (e.g., 60%)^2^. However, because the proportions of carcinoma cells within distinct tumor locations of the same patient are quite different^3^, clinical sampling of macro-dissected cancer tissues could affect the identification of cancer characteristics, including differentially expressed genes (DEGs) and prognostic signatures.

To avoid this uncertainty, several deconvolution algorithms have been proposed to decompose gene expression profiles of macro-dissected samples into cell type-specific subprofiles^4^,^5^, but the requirement of the prior identification of signature genes of pure cells and the measurement of the proportion of cell types limits their application^6^. Another method to tackle this problem involves laser capture microdissection (LCM) technology to acquire a homogeneous collection of thousands of cells that are used to generate cell type-specific gene expression profiles^7^. For example, several researchers have identified DEGs of malignant epithelial cells and stromal cells and analyzed their roles in breast cancer progression^8^,^9^. LCM-coupled microarray studies typically use an additional round of RNA amplification (linear amplification) prior to microarray hybridization because LCM samples are generally too small to yield sufficient mRNA^10^,^11^,^12^,^13^. In some instances, RNA amplification introduces bias in the detection of gene expression values^14^,^15^. However, several studies have provided evidence of a clear correlation between signal intensities resulting from non-amplified mRNA compared with amplified mRNA^16^ with no substantial impact on the identification of DEGs between two groups of LCM samples in the same amplification step^17^.

In this study of estrogen receptor (ER)+ breast cancer patients, we identified DEGs for macro-dissected cancer tissues, malignant epithelial cells and stromal cells, defined as Macro-Dissected-DEGs, Epithelial-DEGs and Stromal-DEGs, respectively, and compared them to reveal the cellular source of Macro-Dissected-DEGs. Then, we evaluated the correlation between expression measurements of DEGs identified in macro-dissected cancer tissues and the proportions of tumor cells in the tissues. Finally, we developed a prognostic signature based on the relative order of gene expression values that commonly occur in malignant epithelial cells and stromal cells compared with normal controls.

## Results

### Comparing Macro-Dissected-DEGs with Epithelial-DEGs and Stromal-DEGs

Using the Rankprod algorithm (see Methods), with 10% FDR control, we extracted DEGs in macro-dissected ER+ breast cancer tissues compared with normal controls from three datasets (M-Data1, M-Data2 and M-Data3, as described in Table 1), respectively. Pairwise comparisons of the three lists of DEGs showed that every two of the DEG lists were significantly overlapped (P-value < 1.0 × 10^−12^, hypergeometric test, see Methods the equation (1)). In addition, the dysregulation consistency scores of the overlapping DEGs of every two DEG lists, defined as the frequency of the overlapping DEGs that showed consistent up- or down-regulation in the two DEG lists, were 83–97%, which were all significantly higher than what expected by chance according to the binomial test (see Methods the equation (2), P-value < 2.2 × 10^−16^, Table S1). These results indicated that the DEGs identified in three independent datasets were significantly reproducible. We extracted DEGs that were dysregulated in the same directions in at least two of the three datasets to construct a list of DEGs that we defined as Macro-Dissected-DEGs.

Using the Rankprod algorithm^18^, with 10% FDR control, we identified two lists of DEGs in malignant epithelial cells compared with normal epithelial cells from two datasets of LCM samples for ER+ breast cancer (Lcm-Data1 and Lcm-Data2, as described in Table1), respectively. The two lists of DEGs contained 547 overlapping DEGs (P-value < 2.2 × 10^−16^, hypergeometric test), among which 97% were dysregulated in the same direction in the two lists. This result indicates that the DEGs of epithelial cells in two independent datasets were significantly reproducible (P-value < 2.2 × 10^−16^, binomial test, Table S1). Given that we could only identify a portion of DEGs in each dataset due to the small sample size^19^, we combined the two lists of DEGs of epithelial cells, deleted DEGs dysregulated in opposite directions, and defined these genes as Epithelial-DEGs. For DEGs identified from the two LCM datasets from stromal cells, 77 DEGs overlapped between the two lists of DEGs (P-value < 2.2 × 10^−16^, hypergeometric test), among which 92% were dysregulated in the same direction (P-value < 2.2 × 10^−16^, binomial test, Table S1). Similarly, we integrated the two lists of DEGs of stromal cells, deleted DEGs dysregulated in opposite directions, and defined these genes as Stromal-DEGs.

Among the 1251 overlapping genes between Epithelial-DEGs and Stromal-DEGs, 86.2% exhibited consistent dysregulation directions (defined as Consistent-DEGs), and the remaining 13.8% were dysregulated in opposite directions (defined as Inconsistent-DEGs). Then, we compared the Consistent-DEGs and Inconsistent-DEGs with Macro-Dissected-DEGs. The consistency score was 90.6% (P-value < 2.2 × 10^−16^, binomial test) among the 790 overlapping genes between Macro-Dissected-DEGs and Consistent-DEGs, which suggested that Consistent-DEGs for both epithelial and stromal cancer cells can be largely reflected in macro-dissected breast cancer tissue. In contrast, among the 91 overlapping genes between Macro-Dissected-DEGs and Inconsistent-DEGs, the consistency score was only 51.7% (P-value = 0.34, binomial test), which suggested that the differential expression signals of such Inconsistent-DEGs, when detected in macro-dissected tissues, were sensitive to the tissue compositions of epithelial and stromal cells. Obviously, the differential expression signals detected in macro-dissected tissues would be consistent with the epithelial DEGs only when the proportion of stromal cell is sufficiently small; otherwise, they would be affected by the stromal cells. Thus, when detected in macro-dissected tissues, the differential expression signals of these Inconsistent-DEGs would be different on datasets of macro-dissected tissues with different composition of epithelial and stromal cells and lack biological interpretation.

### Functional interpretations of Macro-Dissected-DEGs

Based on the biological process (BP) of Gene Ontology (GO), using the GO-function algorithm^20^ designed for selecting non-redundant biologically relevant GO terms from GO terms significantly enriched with DEGs (see Methods), with FDR < 10%, we identified 238 GO terms that were significantly enriched with Macro-Dissected-DEGs. Among the 238 significant terms, 122 terms primarily involved in cell proliferation, developmental growth and cell division tended to be significantly enriched in Consistent-DEGs (P-value < 0.05, hypergeometric test, Table S2). This result suggested that cell proliferation and division processes observed in macro-dissected breast cancer tissue might reflect common alterations among malignant breast epithelial and surrounding stromal cells. Among the 238 significant terms, 18 terms primarily involved in immune responses, biological adhesion and the response to wounding tended to be significantly enriched in Inconsistent-DEGs (P-value < 0.05, hypergeometric test, Table S2). This result indicated that once these immune terms were enriched by Macro-Dissected-DEGs, other evidence was needed to reveal the source of the Macro-Dissected-DEGs.

### The influence of cancer tissue composition on the prognostic signature

For the 376 gene expression profiles extracted from TCGA for ER+ breast cancer tissues which contained 60–100% tumor cell, we evaluated the correlation between the expression measurements of DEGs and the proportions of tumor cell by Pearson correlation analysis (see Methods). The results indicate that, when detected in macro-dissected tissues, the expression levels of 39.8% Consistent-DEGs and 47.8% Inconsistent-DEGs were significantly correlated with the proportions of tumor cell in the macro-dissected cancer tissues (P-value < 0.05, Pearson correlation). Thus, the measurement values of both Consistent-DEGs and Inconsistent-DEGs expression were sensitive to the tissue composition of epithelial and stromal cells.

We extracted the immune signatures developed by *Nagalla et al.*^21^ and *Reyal et al.*^22^ and compared the two lists of signatures with the DEGs identified in our study. The result indicate that some immune signatures were not dysregulated and others were oppositely deregulated in epithelial and stromal cells, and these genes exhibit different dysregulated directions in macro-dissected breast cancer tissues (Table S3). These results demonstrated that immune-associated signatures were greatly affected by clinical cancer tissue sampling. Therefore, we developed a gene pair prognostic signature that was insensitive to the tissue composition of epithelial and stromal cells in macro-dissected breast cancer tissue.

### Prognostic signature based on the relative order of expression

For Lcm-Data1, using the Fisher's exact test, with FDR < 10%, we extracted a list of gene pairs whose relative order of gene expression levels were significantly reversed in malignant epithelial cells compared with normal controls (see Methods). The similar process was performed for stromal cells. These two lists contained 56,268 overlapping gene pairs, among which 99.9% exhibited the same reversal patterns in malignant epithelial and stromal cells compared with normal controls, which was significantly more than expected by chance (P-value < 2.2 × 10^−16^, binomial test). We defined these gene pairs as Consistent-Gene-Pairs. When the Consistent-Gene-Pairs were compared with those extracted from Lcm-Data2, M-Data1, M-Data2 and M-Data1, the consistency scores were all greater than 99.70% (P-value < 2.2 × 10^−16^, binomial test, Table 2), suggesting that Consistent-Gene-Pairs were robust in different datasets.

Based on the integrated raining dataset (Sur-Data1 and Sur-Data2, as described in Table1) for macro-dissected ER+ breast cancer tissues with data of the relapse free survival (RFS), defined as the time period between the date of the first surgery and the date of first relapse, using the univariate Cox model with a FDR < 10%, we identified 17 gene pairs as prognostic gene pairs from the Consistent-Gene-Pairs. For each of the prognostic gene pairs presented in Table 3, the expression level of the latter gene was larger than that of the former gene in patients with better RFS, and the orderings were reversed in patients with worse RFS.

**Figure 1:**
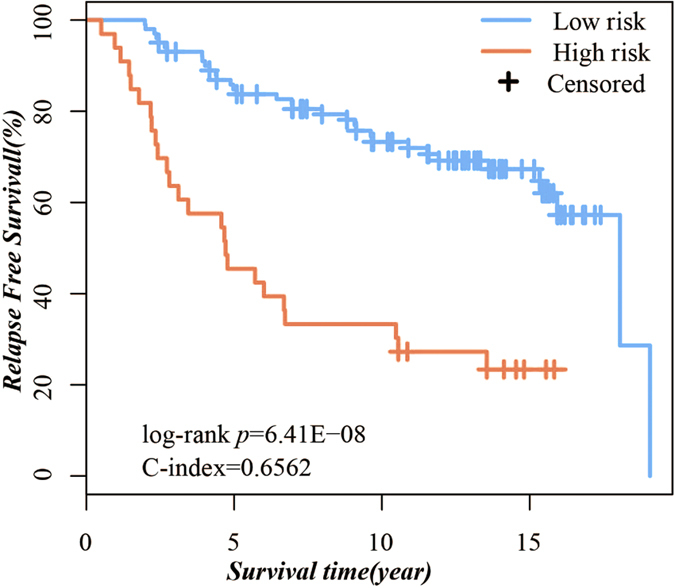
Kaplan–Meier curves illustrating relapse-free survival among patients with ER+ breast cancer based on Prognostic gene pairs in the training set.

According to the classification rule described in the Methods section, the prognostic gene pairs classified the training samples into a high-risk group with 53 samples and a low-risk group with 166 samples, and the RFS of the high-risk patients was significantly reduced compared with low-risk patients (log-rank P *=* 4.15E–10, C-index = 0.66, Fig. 1). After adjusting for grade, age, and tumor size using the multivariate Cox proportional hazards regression model, the prognostic gene pairs were identified as an independent prognostic signature for predicting patient outcomes (Table 4).

**Figure 2:**
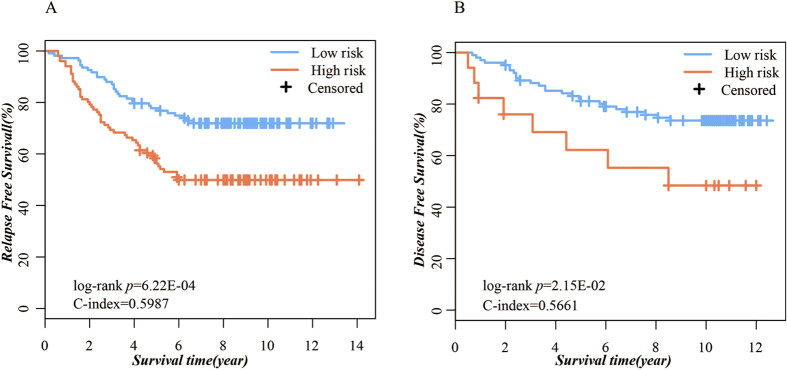
Kaplan–Meier curves illustrating relapse-free survival among patients with ER+ breast cancer based on Prognostic gene pairs in the test sets. (**A**) Test set 1 consisted of Sur-Data3; (**B**) Test set 2 consisted of Sur-Data4.

The accuracy of the prognostic gene pairs was validated in two independent datasets. In Sur-Data3, the prognostic gene pairs classified the 209 patients into 101 high-risk patients and 108 low-risk patients, and the RFS of the high-risk patients was significantly reduced compared with low-risk patients (log-rank P = 9.00E-04, C-index = 0.60, Fig. 2A). For Sur-Data4, disease-free survival (DFS) in 17 high-risk patients was significantly reduced compared with 102 low-risk patients classified by the prognostic gene pairs (log-rank P = 0.03, C-index = 0.57, Fig. 2B). In addition, the prognostic gene pairs were identified as an independent prognostic factor after adjusting for clinical factors, including grade, age, and tumor size using the multivariate Cox proportional hazards regression model in the Sur-Data4 dataset, which contained additional clinical information (Table 4).

## Discussion

The impurity of macro-dissected cancer tissues raises several problems in the analyses of gene expression profiles in cancer tissues. In this study, we demonstrated that most DEGs related to proliferation and division processes observed in breast cancer macro-dissected tissues reflect similar gene expression changes in epithelial and stromal cells, whereas many immune DEGs observed in macro-dissected breast cancer tissues remain controversial. As opposed to epithelial cells, the dysregulation of surrounding stromal cells in breast cancer mainly includes immune-related functions, such as responses to wounds, immune responses and chemotaxis (Table S2 and Table S3). Given the distinct biological processes derived from epithelial and stromal cells, we should be cautious in interpreting DEGs identified from macro-dissected tissues and their related functions. We should also be cautious in interpreting immune related DEGs identified in macro-dissected tissues and micro-dissected stromal cells which include various types of cells, such as leukocytes, endothelial cells, fibroblasts, myofibroblasts and bone marrow-derived progenitors^23^.

Various studies have reported that genes associated with proliferation and immune responses could predict the outcomes of breast cancer patients^22^, and the expressional value of the immune gene prognostic signature is significantly associated with the relative abundance of tumor-infiltrating immune cells^21^. However, the clinical tissue sampling procedure is uncertain, and our present analysis provides evidence that the expression measurements of these prognostic signatures tend to be influenced by the composition of the cancer tissue. To solve this problem, we developed a prognostic gene pairs index based on reversal of the relative order of gene expression values that commonly occur in malignant epithelial cells and stromal cells compared with their normal controls respectively, which is insensitive to the cellular composition of macro-dissected tissues. In addition, the rank-based predictors are more robust than absolute expression value-based predictors because they are rather robust against batch effects and insensitive to data normalization^24^. Furthermore, a rank-based predictor is feasible for individual-level prognostic analysis^25^.

In this study, we focused on breast cancer. It is likely that the same problem exists for other types of tumors; therefore, this subject requires further study.

## Methods

### Data sources and preprocessing

The ten gene expression datasets used in this study were downloaded from the Gene Expression Omnibus (GEO, http://www.ncbi.nlm.nih.gov/geo/)^26^ and The Cancer Genome Atlas (TCGA, http://cancergenome.nih.gov/)^27^. Three macro-dissected ER+ breast tissues with an average tumor cell proportion of approximately 60% were produced by different laboratories^28^,^29^,^30^, and two datasets for malignant epithelial cells and stromal cells of ER+ breast cancer and normal controls were produced by different laboratories^8^,^9^ (Table 1). These datasets were used to identify and compare Macro-Dissected-DEGs, Epithelial-DEGs and Stromal-DEGs. For the ER+ breast cancer data from TCGA^31^, the gene expression profile and the proportion of breast malignant epithelial cells were provided for each sample (Table 1), and these data were used to evaluate the correlation between expression values of DEGs identified in macro-dissected cancer tissues and the proportions of tumor cells in the tissues. Four datasets containing gene expression profiles^32^,^33^,^34^,^35^ and relapse-free survival (RFS) data of ER+ breast cancer patients with early-stage, lymph node negative (LN-) cancer who had not received adjuvant systemic treatment or hormone therapy were used to develop a prognostic signature (Table1).

For the GEO datasets, the raw data (.CEL files) from each dataset was processed using the Robust Multi-array Average (RMA) algorithm for background adjustment with quantile normalization^36^. Then, each probe-set ID was mapped to an Entrez gene ID with the custom CDF file. If multiple probe-sets were mapped to the same gene, the expression value for the gene was summarized as the arithmetic mean of the values of multiple probe-sets. Probe-set IDs with no mapped Entrez gene ID or Probe-set IDs that mapped to more than one Entrez gene ID were deleted. For the TCGA dataset, we applied the level 3 profile directly.

### Identification of differentially expressed genes (DEGs)

The Bioconductor package RankProd^18^, based on the rank products algorithm^37^, was used to identify DEGs of breast cancer versus normal control samples with a false discovery rate (FDR) less than 10%. The P-values were adjusted using the Benjamini-Hochberg procedure^38^. A DEG was considered upregulated (or downregulated) if its average expression level in the cancer samples was increased (or reduced) compared with normal controls.

### Evaluation of the consistency of two DEG lists

If DEG list 1 with *L*_*1*_ genes and DEG list 2 with *L*_2_ genes have *k* overlapping genes, the probability (*P*_*1*_) of observing at least *k* overlapping genes by chance can be calculated according to the following cumulative hypergeometric distribution model:


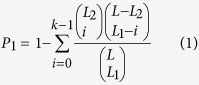


where *L* represents the number of the background genes commonly detected in the datasets from which the DEGs are extracted. The two DEG lists were considered to be significantly overlapping if *P*_*1*_ < 0.05.

If a DEG exhibited the same dysregulated direction (up- or down-regulated) in the two DEG lists, it was considered consistent across the datasets. We defined a dysregulation consistency score as the percentage of consistent DEGs in the overlapping DEGs between the two DEG lists. The probability (*P*_2_) of observing at least *s* DEGs with the same dysregulation direction across the two datasets from *k* randomly selected genes was calculated according to the following cumulative binomial distribution model^39^:


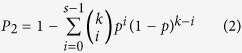


where *p* represents the random possibility (here 0.5) of one DEG having the same dysregulated direction across two DEGs lists. A dysregulation consistency score was considered significant if *P*_2_ < 0.05.

### Functional enrichment analysis

To derive biologically relevant, non-redundant terms from statistically significant terms for a disease, GO-function^20^ was used to select the disturbed functional categories significantly enriched in DEGs. We focused on analyzing the biological process (BP) of Gene Ontology (GO), which was downloaded in April 2013.

### Correlation between the expression measurements of DEGs and the proportions of tumor cell in macro-dissected tissues

Extracted from TCGA, the 376 gene expression profiles for ER+ breast cancer tissues included the data of tumor cell proportions. Using these samples, for the Consistent-DEGs and Inconsistent-DEGs respectively, we applied Pearson’s correlation analysis to detect genes whose expression levels were significantly correlated with tumor cell proportions. Then, the percentages of DEGs that were significantly correlated with tumor cell proportions were calculated for Consistent-DEGs and Inconsistent-DEGs, respectively.

### Development of the prognostic signature based on reversed gene pairs

For a pair of genes, gene A and gene B, we used Fisher’s exact test to evaluate whether the frequency of samples with a higher (or lower) expression level of gene A than gene B in disease samples was significantly different from that in the corresponding normal controls. The P-values were adjusted using the the Benjamini-Hochberg procedure^38^. The significant gene pairs detected with a FDR control level of 10% were defined as significantly reversed gene pairs. Gene pairs with the same reversals of relative ordering of gene expression measurements in malignant epithelial cells and stromal cells were defined as Consistent-Gene-Pairs.

Then, based on the expression profiles of ER+ breast cancer with RFS information, a univariate Cox regression model was used to select gene pairs among the Consistent-Gene-Pairs with a relative order of expression that was significantly correlated with the RFS; these pairs were defined as prognostic gene pairs. The prognostic classifier was constructed according to the following rule: a patient was classified into the low risk group if there were significantly more prognostic gene pairs classifying her as low risk (P-value < 0.05, binomial test); otherwise, the patient was classified into the high risk group. The multivariate Cox proportional hazards regression model was performed to determine whether prognostic gene pairs are an independent prognostic factor in predicting RFS after adjusting for clinical factors, such as age, grade and tumor size.

All statistical analyses were performed using the R 2.15.3 (http://www.r-project.org/).

## Additional Information

**How to cite this article**: Xu, H. *et al.* The influence of cancer tissue sampling on the identification of cancer characteristics. *Sci. Rep.* **5**, 15474; doi: 10.1038/srep15474 (2015).

## Supplementary Material

Supplementary Information

Supplementary Table S2

Supplementary Table S4

Supplementary Table S5

## Figures and Tables

**Table 1:** Summary of the ten datasets analyzed in this study.

Dataset	Sample size		EndPoint	GEO accession ID	Platform
	Cancer	Normal			
Lcm-Data1	30	22	—	GSE14548	U133_X3P
Lcm-Data2	30	10	—	GSE10797	HG-U133A_2
M-data1	28	34	—	GSE10780	HG-U133_Plus_2
M-data2	19	27	—	GSE10810	HG-U133_Plus_2
M-data3	67	17	—	GSE42568	HG-U133_Plus_2
TCGA-Data	376	55	—	—	AgilentG4502A_07
Sur-Data1	134	—	RFS	GSE7390	HG-U133A
Sur-Data2	85	—	RFS	GSE6532	HG-U133A
Sur-Data3	209	—	RFS	GSE2034	HG-U133A
Sur-Data4	119	—	DFS	GSE4922	HG-U133A

Note: Lcm-Data indicates the laser capture microdissection datasets; M-data indicates macro-dissected breast cancer datasets; Sur-Data indicates breast cancer survival datasets; RFS and DFS indicate relapse free survival and disease-free survival, respectively. These datasets were produced by different platforms, including the U133_X3P, HG-U133A_2, HG-U133_Plus_2, AgilentG4502A_07 and HG-U133A platforms, which detected 19703, 12790, 20283, 15621 and 12752 genes, respectively.

**Table 2:** The reproducibility of Consistent-Gene-Pairs.

Dataset	Tis-type	Dis	Nor	Pair-num	Pair-0.05	Direc-con
Lcm-Data2	Lcm_epi	15	5	31271	6372	6353 (99.70%)
Lcm-Data2	Lcm_str	15	5	31271	5762	5748(99.76%)
M-Data1	Tis	28	34	50958	36518	36496(99.94%)
M-Data2	Tis	19	27	50958	29389	29272(99.60%)
M-Data3	Tis	67	17	50958	17204	16547(96.18%)
TCGA-low	Tis	225	55	40025	30806	30438(98.81%)
TCGA-high	Tis	151	55	40025	33114	32790(99.02%)

Note: Lcm and Tis indicate laser capture microdissection datasets and macro-dissected datasets, respectively; Dis and Nor indicate cancer samples and normal controls, respectively; Pair-num indicates the number of gene pairs detected in the datasets; Pair-0.05 indicates the number of gene pairs with a tendency for reversion (P-value < 0.05); Direc-con indicates the number and proportion of gene pairs that are reversed consistently in Pair-0.05 and Com-p; TCGA-low indicates low-tumor purity samples in The Cancer Genome Atlas; TCGA-high indicates high-tumor purity samples in The Cancer Genome Atlas.

**Table 3:** The prognostic gene pairs.

Gene A	Gene B	COX.β	COX.p
CAMLG	KIAA0101	5.0287	7.23E–07
CRIP1	ING1	3.4221	1.17E–05
CRYAB	MAP4K5	3.5943	6.59E–06
CSRP1	RAI14	3.8159	1.65E–06
FBL	PSMD2	0.9705	1.61E–05
FBL	HN1	1.2282	2.20E–05
LMCD1	FGFR4	2.7742	9.62E–06
HOXA4	MAP4K5	2.5518	2.47E–06
SERPINB5	NCAPG	1.1088	1.59E–06
PIGR	KIF4A	0.9484	1.29E–05
PIK3R1	PRC1	0.8993	3.20E–05
SOX10	KIF4A	1.0677	7.40E–07
LMBRD1	KIAA0101	2.2254	2.32E–05
SAV1	KIF4A	1.1495	2.48E–05
OGFRL1	KIF4A	1.1011	3.72E–07
EVL	GPR125	3.5943	6.59E–06
OGFRL1	HJURP	0.8993	3.18E–05

Note: The univariate Cox proportional hazards model was used to estimate the risk coefficient of relative ordering (R_*A*_ > R_*B*_) for each gene pair and the correlation with overall survival in patients; the C-index represents the prognostic performance of relative ordering (R_*A*_ > R_*B*_) for each gene pair.

**Table 4:** Univariate and multivariate Cox regression analysis of the association with RFS.

Characteristic	Subcategory	Univariate analysis		Multivariate analysis	
		HR (95% CI)	P-value	HR (95% CI)	P-value
Training cohort					
Prognostic gene pairs	High vs. low	3.90(2.54–5.98)	4.12E–10	3.60(2.24–5.76)	9.61E–08
Age	>49 vs. ≤49	0.87(0.57–1.32)	0.52	0.95(0.60–1.50)	0.86
Grade	I vs. II, III	1.81(1.01–3.23)	0.04	1.19(0.65–2.18)	0.57
Size	>2 cm vs. ≤2 cm	1.97(1.29–3.00)	1.51E–03	1.74(1.10–2.74)	0.02
Validation cohort					
Prognostic gene pairs	High vs. low	2.45(1.11–5.43)	0.03	2.19(0.95–5.06)	0.06
Age	>66 vs. ≤66	0.7(0.35–1.38)	0.31	0.74(0.37–1.49)	0.41
Grade	I vs. II, III	1.41(0.70–2.86)	0.33	1.12(0.53–2.36)	0.76
Size	>18 mm vs. ≤18 mm	1.78(0.91–3.50)	0.09	1.68(0.85–3.34)	0.14

## References

[b1] Clarke J., Seo P. & Clarke B. Statistical expression deconvolution from mixed tissue samples. Bioinformatics 26, 1043–1049 (2010). 20202973 10.1093/bioinformatics/btq097 PMC2853690

[b2] West M. *et al.* Predicting the clinical status of human breast cancer by using gene expression profiles. P Natl Acad Sci Usa 98, 11462–11467 (2001). 10.1073/pnas.201162998 PMC58752 11562467

[b3] Angell H. & Galon J. From the immune contexture to the Immunoscore: the role of prognostic and predictive immune markers in cancer. Curr Opin Immunol 25, 261–267 (2013). 23579076 10.1016/j.coi.2013.03.004

[b4] Ghosh D. Mixture models for assessing differential expression in complex tissues using microarray data. Bioinformatics 20, 1663–1669 (2004). 14988124 10.1093/bioinformatics/bth139

[b5] Erkkila T. *et al.* Probabilistic analysis of gene expression measurements from heterogeneous tissues. Bioinformatics 26, 2571–2577 (2010). 20631160 10.1093/bioinformatics/btq406 PMC2951082

[b6] Zhao Y. & Simon R. Gene expression deconvolution in clinical samples. Genome Med 2, 93 (2010). 21211069 10.1186/gm214 PMC3025435

[b7] Espina V., Milia J., Wu G., Cowherd S. & Liotta L. A. Laser capture microdissection. Mimb 319, 213–229 (2006). 10.1007/978-1-59259-993-6_10 16719357

[b8] Ma X. J., Dahiya S., Richardson E., Erlander M. & Sgroi D. C. Gene expression profiling of the tumor microenvironment during breast cancer progression. Breast Cancer Res: Bcr 11, R7 (2009). 19187537 10.1186/bcr2222 PMC2687710

[b9] Casey T. *et al.* Molecular signatures suggest a major role for stromal cells in development of invasive breast cancer. Breast Cancer Res Tr 114, 47–62 (2009). 10.1007/s10549-008-9982-8 18373191

[b10] Kube D. M. *et al.* Optimization of laser capture microdissection and RNA amplification for gene expression profiling of prostate cancer. Bmc Mol Biol 8, 25 (2007). 17376245 10.1186/1471-2199-8-25 PMC1847526

[b11] Upson J. J. *et al.* Optimized procedures for microarray analysis of histological specimens processed by laser capture microdissection. J Cell Physiol 201, 366–373 (2004). 15389559 10.1002/jcp.20073

[b12] King C. *et al.* Reliability and reproducibility of gene expression measurements using amplified RNA from laser-microdissected primary breast tissue with oligonucleotide arrays. J Mol Diagn: Jmd 7, 57–64 (2005). 15681475 10.1016/S1525-1578(10)60009-8 PMC1867505

[b13] van Haaften R. I. *et al.* Biologically relevant effects of mRNA amplification on gene expression profiles. Bmc Bioinformatics 7, 200 (2006). 16608515 10.1186/1471-2105-7-200 PMC1523219

[b14] de Bruin E. C. *et al.* Macrodissection versus microdissection of rectal carcinoma: minor influence of stroma cells to tumor cell gene expression profiles. Bmc Genomics 6, 142 (2005). 16225673 10.1186/1471-2164-6-142 PMC1283972

[b15] Michel C. *et al.* Liver gene expression profiles of rats treated with clofibric acid: comparison of whole liver and laser capture microdissected liver. Am J Pathol 163, 2191–2199 (2003). 14633594 10.1016/S0002-9440(10)63577-8 PMC1892366

[b16] Schneider J. *et al.* Systematic analysis of T7 RNA polymerase based *in vitro* linear RNA amplification for use in microarray experiments. Bmc Genomics 5, 29 (2004). 15119961 10.1186/1471-2164-5-29 PMC419340

[b17] Klee E. W. *et al.* Impact of sample acquisition and linear amplification on gene expression profiling of lung adenocarcinoma: laser capture micro-dissection cell-sampling versus bulk tissue-sampling. Bmc Med Genomics 2, 13 (2009). 19272143 10.1186/1755-8794-2-13 PMC2667433

[b18] Hong F. *et al.* RankProd: a bioconductor package for detecting differentially expressed genes in meta-analysis. Bioinformatics 22, 2825–2827 (2006). 16982708 10.1093/bioinformatics/btl476

[b19] Zhang M. *et al.* Apparently low reproducibility of true differential expression discoveries in microarray studies. Bioinformatics 24, 2057–2063 (2008). 18632747 10.1093/bioinformatics/btn365

[b20] Wang J. *et al.* GO-function: deriving biologically relevant functions from statistically significant functions. Brief Bioinform 13, 216–227 (2012). 21705405 10.1093/bib/bbr041

[b21] Nagalla S. *et al.* Interactions between immunity, proliferation and molecular subtype in breast cancer prognosis. Genome Biol 14, R34 (2013). 23618380 10.1186/gb-2013-14-4-r34 PMC3798758

[b22] Reyal F. *et al.* A comprehensive analysis of prognostic signatures reveals the high predictive capacity of the proliferation, immune response and RNA splicing modules in breast cancer. Breast Cancer Res: Bcr 10, R93 (2008). 19014521 10.1186/bcr2192 PMC2656909

[b23] Horimoto Y., Polanska U. M., Takahashi Y. & Orimo A. Emerging roles of the tumor-associated stroma in promoting tumor metastasis. Cell Adhes Migr 6, 193–202 (2012). 10.4161/cam.20631 PMC3427234 22568980

[b24] Zhou X. *et al.* A relative ordering-based predictor for tamoxifen-treated estrogen receptor-positive breast cancer patients: multi-laboratory cohort validation. Breast Cancer Res Tr 142, 505–514 (2013). 10.1007/s10549-013-2767-8 24253811

[b25] Wang H. *et al.* Individual-level analysis of differential expression of genes and pathways for personalized medicine. Bioinformatics 31, 62–68 (2015). 25165092 10.1093/bioinformatics/btu522

[b26] Barrett T. *et al.* NCBI GEO: mining tens of millions of expression profiles--database and tools update. Nucleic Acids Res 35, D760–765 (2007). 17099226 10.1093/nar/gkl887 PMC1669752

[b27] Cancer Genome Atlas N. Comprehensive molecular portraits of human breast tumours. NATURE 490, 61–70 (2012). 23000897 10.1038/nature11412 PMC3465532

[b28] Chen D. T. *et al.* Proliferative genes dominate malignancy-risk gene signature in histologically-normal breast tissue. Breast Cancer Res Tr 119, 335–346 (2010). 10.1007/s10549-009-0344-y PMC2796276 19266279

[b29] Pedraza V. *et al.* Gene expression signatures in breast cancer distinguish phenotype characteristics, histologic subtypes, and tumor invasiveness. Cancer 116, 486–496 (2010). 20029976 10.1002/cncr.24805

[b30] Clarke C. *et al.* Correlating transcriptional networks to breast cancer survival: a large-scale coexpression analysis. Carcinogenesis 34, 2300–2308 (2013). 23740839 10.1093/carcin/bgt208

[b31] Michailidou K. *et al.* Large-scale genotyping identifies 41 new loci associated with breast cancer risk. Nat Genet 45, 353–361, 361e351-352 (2013). 23535729 10.1038/ng.2563 PMC3771688

[b32] Desmedt C. *et al.* Strong time dependence of the 76-gene prognostic signature for node-negative breast cancer patients in the TRANSBIG multicenter independent validation series. Clin Cancer Res: an official journal of the American Association for Cancer Research 13, 3207–3214 (2007). 10.1158/1078-0432.CCR-06-2765 17545524

[b33] Loi S. *et al.* PIK3CA mutations associated with gene signature of low mTORC1 signaling and better outcomes in estrogen receptor-positive breast cancer. P Natl Acad Sci Usa 107, 10208–10213 (2010). 10.1073/pnas.0907011107 PMC2890442 20479250

[b34] Wang Y. *et al.* Gene-expression profiles to predict distant metastasis of lymph-node-negative primary breast cancer. Lancet 365, 671–679 (2005). 15721472 10.1016/S0140-6736(05)17947-1

[b35] Ivshina A. V. *et al.* Genetic reclassification of histologic grade delineates new clinical subtypes of breast cancer. Cancer Res 66, 10292–10301 (2006). 17079448 10.1158/0008-5472.CAN-05-4414

[b36] Irizarry R. A. *et al.* Exploration, normalization, and summaries of high density oligonucleotide array probe level data. Biostatistics 4, 249–264 (2003). 12925520 10.1093/biostatistics/4.2.249

[b37] Breitling R., Armengaud P., Amtmann A. & Herzyk P. Rank products: a simple, yet powerful, new method to detect differentially regulated genes in replicated microarray experiments. Febs Lett 573, 83–92 (2004). 15327980 10.1016/j.febslet.2004.07.055

[b38] Hochberg Y. B. A. Y. Controlling the False Discovery Rate: A Practical and Powerful Approach to Multiple Testing. J R Stat Soc B 57, 289–300 (1995).

[b39] Bahn A. K. Application of binomial distribution to medicine: comparison of one sample proportion to an expected proportion (for small samples). Evaluation of a new treatment. Evaluation of a risk factor. Am J Med Genet A 24, 957–966 (1969). 4243043

