# Supplementary material for: The influence of cancer tissue sampling on the identification of cancer characteristics
Source: Sci Rep. 2015 Oct 22;5:15474. doi: 10.1038/srep15474 (PMC4614546; doi:10.1038/srep15474)
Supplement: Supplementary Information [file srep15474-s1.pdf]

# The influence of cancer tissue sampling on the identification of cancer characteristics

Hui Xu[1], Xin Guo[1], Qiang Sun[2], Mengmeng Zhang[1], Lishuang Qi[1], Yang Li[1], Libin Chen[1], Yunyan Gu[1], Zheng Guo[1, 3*], Wenyuan Zhao[1*]

[1]College of Bioinformatics Science and Technology, Harbin Medical University, Harbin, 150086, China;

[2]Genomics Research Center, Harbin Medical University, Harbin, 150086, China;

[3]Key Laboratory of Ministry of Education for Gastrointestinal Cancer, Department of Bioinformatics, Fujian Medical University, Fuzhou, 350004, China

[*] Corresponding authors

# Supplementary Tables

**Table S1. The reproducibility of DEGs derived from different datasets**

| Dataset1 vs. Dataset2 | DEG1 | DEG2 | Overlap | Consistency |
|---|---|---|---|---|
| M-data1 vs. M-data2 | 5861 | 5573 | 3505 | 92% |
| M-data1 vs. M-data3 | 5861 | 6129 | 3367 | 82.7% |
| M-data2 vs. M-data3 | 5573 | 6129 | 3818 | 96.8% |
| Lcm-data1 vs. Lcm-data2(epi) | 2848 | 547 | 332 | 97% |
| Lcm-data1 vs. Lcm-data2(str) | 2116 | 162 | 77 | 92% |

*Abbreviations:* ***DEG1****,* ***DEG2,*** *the numbers of DEGs derived from Dataset1 and Dataset2, respectively；* ***Overlap,*** *the number of overlapped DEGs between DEG1 and DEG2;* ***Consistency,*** *the dysregulation consistency percentage of overlapped DEGs.*

**Table S3. The comparison of immune signatures and DEGs derived from our study**

| Immune signatures | Lcm-Data | | M-Data | |
|---|---|---|---|---|
| | In-DEGs | DEGs (Percent) | In-DEGs | DEGs (Percent) |
| *Nagalla* et al. | 58 | 42 (41.6%) | 39 | 120 (42.5%) |
| *Reyal* et al. | 38 | 43 (30.2%) | 13 | 68 (22.05%) |

*Abbreviations:* ***Immune signatures*** *represent the immune related gene signature extracted from Nagalla* et al. *and Reyal* et al.; ***In-DEGs*** *represent genes with no differential tendency between breast cancer and normal controls;* ***Percent*** *represents the percentage of* ***DEGs*** *that were dysregulated in consistent directions.*
